# Hypoxemia secondary to thymoma excision with 1-lung ventilation: A case report

**DOI:** 10.1097/MD.0000000000035373

**Published:** 2024-01-19

**Authors:** Hao Xu

**Affiliations:** a Department of Critical Care Medicine, Wuxi No. 2 People’s Hospital, Wuxi Jiangsu, China.

**Keywords:** acute respiratory distress syndrome, hypoxemia, one-lung ventilation, prone positioning

## Abstract

**Introduction:**

Acute respiratory distress syndrome (ARDS) is an acute respiratory illness characterized by bilateral chest radiographic opacities and severe hypoxemia due to noncardiogenic pulmonary edema. However, ARDS due to sing lung injury is rare. In this article, we describe a case of a female patient who developed ARDS during surgery and 1-lung mechanical ventilation.

**Methods:**

A 53-year-old woman developed hypoxemia 2 days after undergoing thymoma excision. Antibiotics, diuretics, expectorants, and supportive treatment strategies (noninvasive positive-pressure breathing, high-flow nasal oxygen) were used for approximately 3 days after admission to the intensive care unit (ICU), but the patient’s oxygen index continued to deteriorate. Considering the possibility of ARDS being induced by 1-lung mechanical ventilation, prone positioning, methylprednisolone, nintedanib, and acetylcysteine were administered.

**Result:**

Oxygenation improves greatly after 23 days admitted to ICU. Chest computed tomography shows a real reversal of the disease. The patient was discharged from ICU 29 days after admission to the ICU and was discharged from the hospital after 36 days admitted to ICU.

**Conclusion:**

In this report, we describe a rare case of ARDS involved 1 lung, in which we successfully used noninvasive positive-pressure breathing, high-flow nasal oxygen, and prone positioning to succeed improvement of clinical outcomes. The use of the prone position has benefits in nonintubated patient with ARDS even involved 1 lung.

## 1. Introduction

Acute respiratory distress syndrome (ARDS) is a life-threatening form of respiratory failure, which can be caused by a variety of pulmonary (e.g., pneumonia, aspiration) or nonpulmonary (e.g., sepsis, pancreatitis, trauma) insults, leading to the development of nonhydrostatic pulmonary edema.^[1]^ The prevalence of ARDS in patients in intensive care was 10%, and mortality in the subgroup of patients with severe ARDS was 46%.^[2]^ There has been a renewed focus on the potential for mechanical ventilation to cause harm. Ventilator-induced lung injury occurs most readily in patients with concomitant physiological insults (e.g., sepsis, trauma, major surgery) that prime the immune system for a cascading response to mechanical lung injury.^[3]^ This article describes the clinical data related to the diagnosis and treatment process of 1 patient developed ARDS during operation and 1-lung mechanical ventilation to further improve the diagnosis and treatment of this type of disease.

## 2. Case presentation

**Figure 1.**
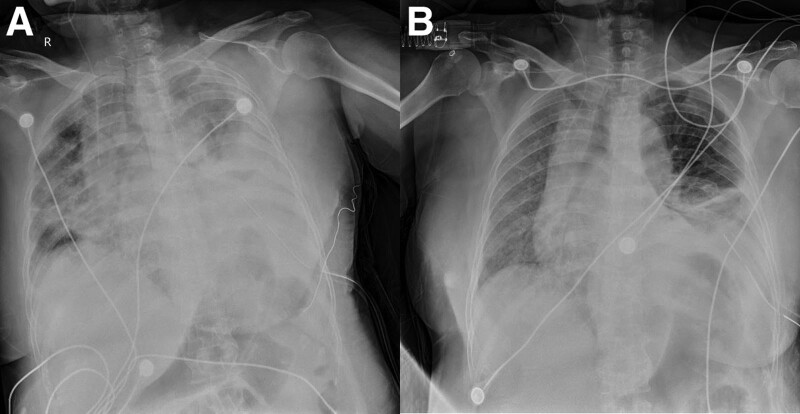
Chest radiographs of the patient. Initial supine chest radiography obtained on presentation to the ICU (A) shows pulmonary edema, left pleural effusion. A supine anteroposterior portable chest radiograph obtained 3 d after presentation to the ICU (B) shows left atelectasis and pleural effusion and right alveolar infiltrate. ICU = intensive care unit.

A 53-year-old woman was admitted to the intensive care unit (ICU) of our hospital for hypoxemia. On the morning before admitted to the ICU, dyspnea occurred at rest. That afternoon, hypoxemia worsened; the patient was receiving oxygen through a face mask at a rate of 10 L per minute, and the respiratory rate was 30 breaths/min, then she was admitted to the ICU. On presentation to the ICU, a physical examination revealed the following: temperature, 36.5; heart rate, 80 breats/min; respiratory rate, 25 breaths/min (while he was receiving oxygen by oral-nasal mask); and BP, 133/82 mm Hg. Results of laboratory tests showed the following: WBC count, 10,090/μL with 94.7% neutrophils; hemoglobin, 10.0 g/dL; platelets, 146 × 104/μL; oxygen index was 171 mm Hg (Table 1). Rapid antigen testing for influenza types A and B was negative. Initial supine chest radiography (Fig. 1A) shows pulmonary edema, left pleural effusion. Echocardiography reveals a mildly enlarged left atrium with a good left cardiac function (62%) and mild tricuspid regurgitation.

**Table 1:** Laboratory data.

Variable	Reference range	On presentation, this hospital	On presentation to the ICU	Day 6, admission in ICU
Venous blood				
Hemoglobin (g/dL)	11.5–15.0	13.3	10.6	11.8
Hematocrit (%)	35.0–45.0	39.0	31.0	34.0
White cell count (10^9^/L)	3.5–9.5	4.6	10.9	7.9
Differential count (%)				
Neutrophils	40–75	73	97	89
Lymphocytes	20–50	20	2	7
Monocytes	4–11	7	0.1	0.3
Eosinophils	0–8	0	0.1	0.1
Basophils	0–3	0.3	0	0.1
Platelet count (10^9^/L)	125–350	203	146	267
Lymphocytes subpopulations				
CD16 CD56 (%)	10.0–19.8	4.8	/	/
CD19 (%)	9.0–14.1	11.7	/	/
CD3 (%)	64.2–75.8	77.2	/	/
CD4 (%)	30.1–40.4	46.1	/	/
CD8 (%)	25.7–29.4	25.1	/	/
CD4/CD8	1.0–1.9	1.8	/	/
CD45	100	100	/	/
Sodium (mmol/L)	136.0–145.0	140.5	137.6	135.4
Potassium (mmol/L)	3.5–5.3	4.1	3.9	3.3
Chloride (mmol/L)	96.0–108.0	105.4	104.0	91.9
Urea nitrogen (mmol/L)	2.1–8.6	3.7	5.4	9.8
Creatinine (μmol/L)	35.2–97.5	47.5	55.4	51.9
Glucose (mmol/L)	3.8–6.2	4.2	5.8	4.8
Calcium (mmol/L)	2.1–2.7	2.2	2.2	2.1
Total protein (g/L)	55.0–82.0	68.5	58.6	67.2
Albumin (g/L)	35.0–55.0	37.9	31.1	41.5
Globulin (g/L)	18.0–35.0	30.6	27.5	25.7
Total bilirubin (μmol/L)	3.4–20.6	9.3	11.9	14.3
Aspartate aminotransferase (U/L)	5–50	15.3	16	31
Alanine aminotransferase (U/L)	5–50	12.4	12	11
Alkaline phosphatase (U/L)	82	129.4	37–123	241
Erythrocyte sedimentation rate (mm/h)	0–20.0	30	117.0	90.0
C-reactive protein (mg/L)	0–10	5	120	95
Procalcitonin (ng/L)	0–0.5	/	0.3	/
Lactate dehydrogenase (U/L)	109–245	255	160	347
γ-glutamyl transferase (U/L)	8–58	19	18	122
C3 (g/L)	0.6–1.6	/	1.1	/
C4 (g/L)	0.1–0.5	/	0.2	/
Arterial blood gas				
Fraction of inspired oxygen	/	0.2	1.0	1.0
pH	7.35–7.45	7.40	7.49	7.52
Partial pressure of carbon dioxide (mm Hg)	35–45	44	34	43
Partial pressure of oxygen (mm Hg)	80–100	96	171	123
Lactate (mmol/L)	0.5–1.6	/	1.3	1.1
Pro-BNP (pg/mL)	0–900	/	603	/

ICU = intensive care unit.

The oxygen saturation increased to 98% while he was receiving noninvasive positive-pressure ventilation (fraction of inspired oxygen = 0.6), and the respiratory rate decreased to 25 breaths/min. Furosemide, lyophilized recombinant, and omeprazole were administered. Initial supine chest radiography obtained on presentation to the ICU (Fig. 1A) shows pulmonary edema, left pleural effusion. A supine chest radiograph obtained 3 days after presentation to the ICU (Fig. 1B) shows left atelectasis and pleural effusion and right alveolar infiltrate.

**Figure 2.**
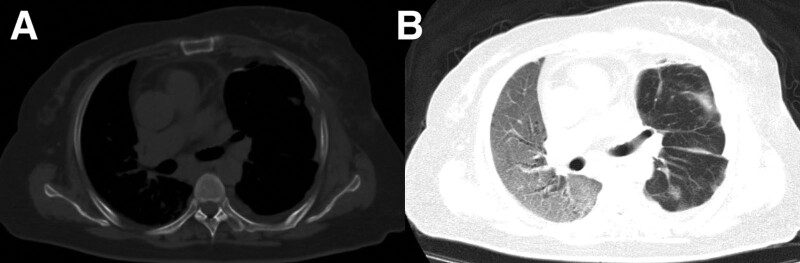
CT imagines of the patient obtained 5 d after presentation to the ICU shows diffuse infiltration of the right lung. CT = computed tomography, ICU = intensive care unit.

The cumulative negative fluid balance after 3 days of ICU stay was 6 L, but oxygen index was just 144 mm Hg while he was receiving noninvasive positive-pressure ventilation or high-flow nasal cannula oxygen therapy. A repeat chest radiograph (Fig. 1B) shows left atelectasis and pleural effusion and right alveolar infiltrate. Repeated examination of infection indicators and sputum culture were all negative. Prone position was performed for 1 to 2 hours (since poor compliance of patient) every day. Five days after admission in the ICU, chest computed tomography (CT) shows diffuse infiltration of the right lung (Fig. 2).

**Figure 3.**
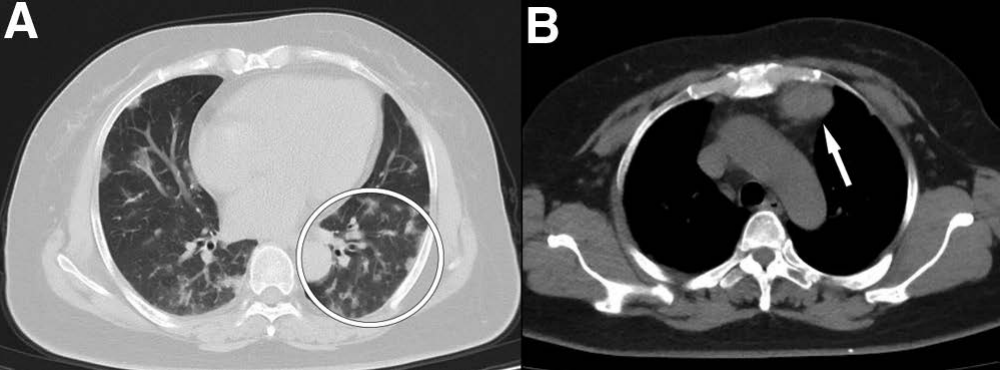
Chest CT imaging obtained 1 wk before admission. Axial images show bilateral multiple patchy high-density shadows and fibrous foci (A, oval) and space occupying lesions in mediastinum (B). CT = computed tomography.

**Figure 4.**
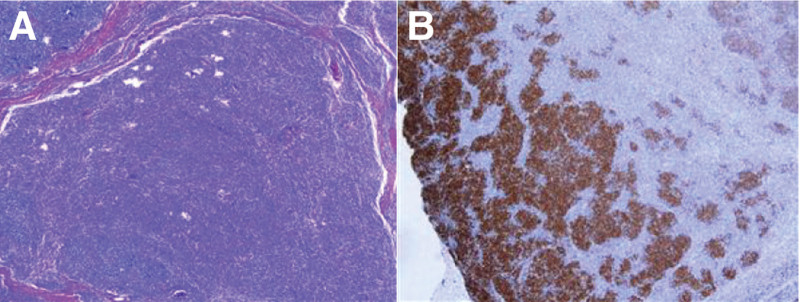
A biopsy of thymus. A biopsy of thymus shows type AB thymoma (A), and there were large quantity of TdT-positive T-cells in tumorous tissues (B). Immunolabeling of tumor cell show CD117 (−), CK (pan) (+), EMA (+), CK14 (+), CK7 (−), Ki-67 (<5%). Immunolabeling of lymphoid tissue show CD la (+), CD5 (+), CD45 (LCA) (+), Ki-67 (+80%).

She came to our hospital due to dry cough and abnormal findings on chest CT (Fig. 3). Three days before admission in ICU, video-assisted excision of thymoma, mediastinal lymph node dissection, and left inferior wedge-shaped lobectomy (since palpable multiple hard lung nodules in the operation) was performed. One-lung ventilation with a double-lumen tube has been used to guarantee good surgical exposure. Mechanical ventilation during 1-lung ventilation was initiated using volume-controlled ventilation mode with a peak airway pressure below 30 cm H_2_O (tidal volume was 250–300 mL, PEEP was 5 cm H_2_O, fraction of inspired oxygen was 1.0) and a respiratory rate of 18 breaths/min. A biopsy of specimen was performed, the anatomopathological examination concluded to type AB thymoma (Fig. 4). She had no known allergies to medications. She did not smoke tobacco, drink alcohol, or use illicit drugs. She worked at a wet market. She had no history of dust exposure. She had undergone cholecystectomy, hysteromyomectomy, and surgery for lower limb fracture. There was no other family history of cancer.

## 3. Discussion

There was no doubt that primary diagnosis was thymoma of type AB. Thymomas account for 47% tumors of anterior mediastinum. They occur in all ages, but there is a broad peak between 35 and 70 years of age. Thymoma could be asymptomatic (30%) and discovered incidentally including chest surgery for unrelated reasons. About 40% of patients have local symptoms. They usually include chest pain, cough, and shortness of breath from compression of the airways or from the neuromuscular effects of myasthenia gravis. CT scan is highly sensitive in diagnosis and certain features can predict the presence of advanced stage disease. Excellent 5- and 10-year survival rates are noted for completely resected early stage thymomas. Complete resection is the best prognostic factor for these tumors.^[4]^

The second diagnosis was ARDS (right lung). ARDS is characterized by an acute, diffuse, inflammatory lung injury, leading to increased alveolar capillary permeability, increased lung weight, and loss of aerated lung tissue.^[5]^ Clinically, this manifests as hypoxemia, with bilateral opacities on chest radiography, associated with decreased lung compliance and increased venous admixture and physiological dead space. Morphologically, diffuse alveolar damage is seen in the acute phase of ARDS. The Berlin definition of ARDS^[5]^ are following: within 1 week of a known clinical insult or new or worsening respiratory symptoms; bilateral opacities—not fully explained by effusions, lobar/lung collapse, or nodules; respiratory failure not fully explained by cardiac failure or fluid overload; oxygen index ≤300 mm Hg with PEEP or CPAP ≥5 cm H_2_O.

One-lung ventilation exposes to the risk of postoperative acute lung injury. The incidence of ARDS after thoracic surgery was 2% with a mortality of 54.5%.^[6]^ A retrospective cohort study^[7]^ on patients undergoing surgery for lung cancer revealed that the risk factors for the primary ARDS manifested in the first 3 postoperative days included chronic alcohol consumption, pneumonectomy, high inspiratory pressure during mechanical ventilation, and fluid overload. Other known risk factors are the duration of 1-lung ventilation, severe pulmonary dysfunction, neoadjuvant chemotherapy, and multiple transfusions.^[8]^

This 53-year-old woman presented with hypoxemia and diffuse infiltrate of right lung in 3 days after operation with 1-lung ventilation. Echocardiography ruled out pulmonary edema due to cardiac insufficiency. Chest CT revealed no effusions, lobar/lung collapse, and nodules. Oxygen index was still below 150 mm Hg through negative fluid balance. Although protective ventilation with small tidal volume was performed, 1-lung ventilation caused acute lung injury of right lung. The left lung avoided ventilator-induced lung injury due to a collapse of lung to guarantee good surgical exposure.

## 4. Pregnosis

All thymomas should be resected due to their malignant potential, providing patients are otherwise healthy. An attempt should be made to perform a complete resection of thymic cells. Tumor stage and completeness of resection are the 2 most important predictors of survival. Presence of myasthenia gravis is not an independent prognostic factor but may play a role in early detection of thymic tumors.^[4]^

The American Thoracic Society, European Society of Intensive Care Medicine, and Society of Critical Care Medicine have endorsed clinical practice guidelines on mechanical ventilation in adult patients with ARDS.^[9]^ The guidelines provide clinical recommendations on 6 interventions including strong recommendations for the use of volume-limited and pressure-limited ventilation and prone positioning for more than 12 h/d in patients with severe ARDS; a strong recommendation against the routine use of HFOV; conditional recommendations for the use of lung recruitment maneuvers and high PEEP strategies in patients with moderate or severe ARDS; and insufficient data to make a recommendation for or against venovenous extracorporeal membrane oxygenation in patients with severe ARDS.

**Figure 5.**
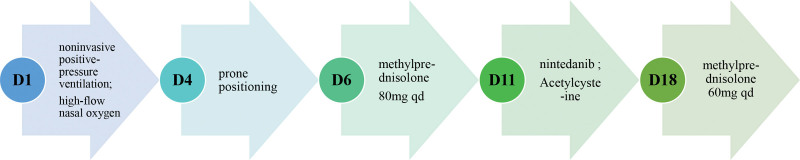
Therapeutic schedule. D1 = the date of admission in ICU, D4 = three days after admission in ICU, and so forth, qd = quaque die.

**Figure 6.**
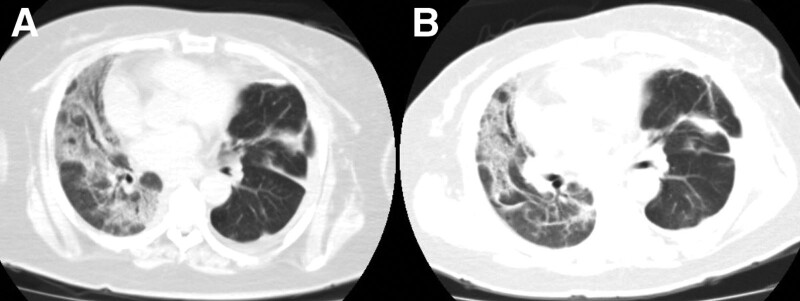
Chest CT imaging. Axial images (A, B) obtained 22 and 29 d after admission in ICU show absorption of lesions. CT = computed tomography, ICU = intensive care unit.

The patient presented with a rare case of ARDS involved 1 lung. We didn’t perform endotracheal intubation due to absence of severe respiratory distress. What’s more, invasive mechanical ventilation may well be unable to protect damaged lung since we can’t get drive pressure of right lung. We maintain patient’s oxygenation through alternative use of noninvasive positive-pressure ventilation and high-flow nasal oxygen (Fig. 5). Methylprednisolone, nintedanib, and acetylcysteine were administered to yield an antifibrotic benefit. Prone positioning was performed to improve lung compliance. Oxygen index and chest CT improved (Fig. 6), and the patient was discharged from ICU 29 days after admission to the ICU, and was discharged from hospital 36 days after admission to the ICU.

## 5. Conclusions

In this report, we describe a rare case of ARDS involving 1 lung, in which we successfully used noninvasive positive-pressure breathing, high-flow nasal oxygen and prone positioning to succeed improvement of clinical outcomes. The use of the prone position has benefit in nonintubated patient with ARDS even involved 1 lung.

## Author contributions

**Writing – original draft:** Hao Xu.
